# Lentiviral Gene Transfer Corrects Immune Abnormalities in XIAP Deficiency

**DOI:** 10.1007/s10875-022-01389-0

**Published:** 2022-11-03

**Authors:** Joseph Topal, Neelam Panchal, Amairelys Barroeta, Anna Roppelt, Annelotte Mudde, H. Bobby Gaspar, Adrian J. Thrasher, Benjamin C. Houghton, Claire Booth

**Affiliations:** 1 Molecular and Cellular Immunology Section, UCL Great Ormond Street Institute of Child Health, London, UK; 2 Dmitry Rogachev National Medical Research Center of Pediatric Hematology, Oncology and Immunology, Moscow, Russian Federation; 3 Orchard Therapeutics, London, UK; 4 Department of Immunology and Gene Therapy, Great Ormond Street Hospital for Children, NHS Foundation Trust, London, UK

**Keywords:** XIAP, XIAP deficiency, X-linked inhibitor of apoptosis, hematopoietic stem cell gene therapy, NOD2, dectin-1

## Abstract

**Background:**

X-linked inhibitor of apoptosis protein (XIAP) deficiency is a severe immunodeficiency with clinical features including hemophagocytic lymphohistiocytosis (HLH) and inflammatory bowel disease (IBD) due to defective NOD2 responses. Management includes immunomodulatory therapies and hematopoietic stem cell transplant (HSCT). However, this cohort is particularly susceptible to the chemotherapeutic regimens and acutely affected by graft-vs-host disease (GvHD), driving poor long-term survival in transplanted patients. Autologous HSC gene therapy could offer an alternative treatment option and would abrogate the risks of alloreactivity.

**Methods:**

Hematopoietic progenitor (Lin^−ve^) cells from XIAP^y/−^ mice were transduced with a lentiviral vector encoding human XIAP cDNA before transplantation into irradiated XIAP ^y/−^ recipients. After 12 weeks animals were challenged with the dectin-1 ligand curdlan and recovery of innate immune function was evaluated though analysis of inflammatory cytokines, body weight, and splenomegaly. XIAP patient-derived CD14^+^ monocytes were transduced with the same vector and functional recovery was demonstrated using in vitro L18-MDP/NOD2 assays.

**Results:**

In treated XIAP^y/−^ mice, ~40% engraftment of gene-corrected Lin^−ve^ cells led to significant recovery of weight loss, splenomegaly, and inflammatory cytokine responses to curdlan, comparable to wild-type mice. Serum IL-6, IL-10, MCP-1, and TNF were significantly reduced 2-h post-curdlan administration in non-corrected XIAP^y/−^ mice compared to wild-type and gene-corrected animals. Appropriate reduction of inflammatory responses was observed in gene-corrected mice, whereas non-corrected mice developed an inflammatory profile 9 days post-curdlan challenge. In gene-corrected patient CD14^+^ monocytes, TNF responses were restored following NOD2 activation with L18-MDP.

**Conclusion:**

Gene correction of HSCs recovers XIAP-dependent immune defects and could offer a treatment option for patients with XIAP deficiency.

**Supplementary Information:**

The online version contains supplementary material available at 10.1007/s10875-022-01389-0.

## Introduction

X-linked inhibitor of apoptosis protein (XIAP) deficiency is a primary immunodeficiency caused by mutations in the XIAP gene, and is estimated to occur in 1–2 per million live births [1, 2]*.* XIAP deficiency was first described in 2006 in association with recurrent hemophagocytic lymphohistiocytosis (HLH), now additional manifestations are being frequently recognized including inflammatory bowel disease (IBD), hypogammaglobulinemia, severe recurrent infections, autoimmunity, cytopenias, and other inflammatory complications [1, 3–6].

Given the range of clinical symptoms and disease course, there are no standardized treatment guidelines, and thus therapeutic intervention is primarily guided by clinical manifestations and includes immunosuppression and immunomodulatory drugs [7]. Currently, the only curative treatment option for XIAP deficiency is allogeneic hematopoietic stem cell transplantation (HSCT). In patients with severe disease, including HLH and severe refractory IBD, HSCT is often considered. However, early reports demonstrated poor transplant outcomes in this disease, with long-term survival below 50% [8]. Myeloablative conditioning (MAC) regimens were associated with poor long-term survival, (43% 5-year overall survival) particularly in the context of HLA-mismatched donors or ongoing HLH at time of transplantation [8]. More recent reports of reduced-intensity conditioning (RIC) regimens suggest these approaches are better tolerated with improved survival [8–10]. However, HSCT is associated with graft-versus-host disease (GvHD), which significantly increases the risk of mortality in patients with XIAP deficiency [9]. Autologous hematopoietic stem cell (HSC) gene therapy abrogates any risk of alloreactivity due to GvHD making it an extremely attractive potential therapy. For over 20 years, autologous HSC gene therapy has been a promising treatment option for specific immune disorders [11–16], and this success continues to promote the development of new gene therapy approaches for other monogenic immune disorders including several forms of familial HLH [17–20].

XIAP is an antiapoptotic protein that regulates cell death in response to apoptotic stimuli by directly inhibiting caspase-3, caspase-7, and caspase-9 via its N-terminal baculoviral IAP repeat (BIR) domains [21–24]. As such, T cells from XIAP deficient patients, including invariant natural killer T cells (iNKT) and mucosal-associated invariant T (MAIT) cells which express elevated levels of caspases that are inhibited by XIAP, have an increased sensitivity to activation-induced cell death (AICD) [1, 5, 6, 25]. Although blood lymphocyte cell numbers may be within normal ranges [2], it is thought that suboptimal expansion of virus-specific T cells occurs following infection in XIAP deficient patients [2, 5–7]. XIAP is also involved in other immune signaling pathways essential for innate immune responses. The RING and BIR2 domains of XIAP are required for nuclear factor-κB (NF-κB) and mitogen-activated protein kinase (MAPK) signaling in response to activation of nucleotide-binding oligomerization domain 1 (NOD1) and NOD2 [26, 27]. Several studies have shown that peripheral monocytes from XIAP-deficient patients have compromized cytokine production, including TNF and IL-8, in response to NOD2 ligands, while TLR-2 and TLR-4 receptor stimulation is unaffected [3, 28, 29]. Impaired NOD2 signaling is implicated in the development of IBD in XIAP deficiency. In addition, serum levels of pro-inflammatory cytokines, including IL-6, IL-2, IFN-γ, and TNF are elevated in XIAP-deficient patients with HLH [30]. IL-18 levels are significantly elevated during HLH in XIAP deficiency and remain chronically elevated during remission [30, 31].

XIAP is known to play a role in dectin-1 signaling, a transmembrane pattern recognition receptor (PRR) involved in antifungal immunity through β-glucan recognition [32, 33]. The observations by Hsieh et al. demonstrate that XIAP-deficient mice have impaired innate immune responses following dectin-1 priming, which result in XIAP deficiency-like features [32]. XIAP is also an important regulator of the NLRP3 inflammasome. Studies show loss of XIAP result in dysregulated caspase-1/NLRP3 inflammasome activation, which is associated with overproduction of pro-inflammatory cytokines and cell death [25, 34–36]. Furthermore, XIAP prevents TNF-mediated, receptor-interacting protein 3 (RIPK3)-dependent cell death, by regulating RIPK1 ubiquitylation, and inhibiting inflammatory cell death [34].

Due to the role of XIAP in preventing cell death, over-expression of XIAP can enhance cell tolerance to external and internal apoptotic stimuli [37], and dysregulation of XIAP has been shown to contribute toward the progression of multiple cancers, including bladder [38–40], breast [41–43], ovarian [44–46], lung [47–49], colon [50–52], and prostate [53–56]. Therefore, the expression profile of XIAP is thought to be part of a system of regulatory loops that balance a cell’s response to environmental stimuli [37, 57]. In addition to its anti-apoptotic function, extensive studies suggests that XIAP is important for both the clearance of pathogens and the regulation of inflammatory responses.

To address the treatment challenges in this condition, we generated a self-inactivating lentiviral vector containing human XIAP cDNA under the control of a spleen focus forming virus (SFFV) promoter, as well as a GFP reporter gene (LV-SFFV-XIAP-GFP) and investigated whether gene transfer could correct immune abnormalities and phenotype in a murine model of the disease and XIAP-deficient patient cells. Here for the first time, we show that lentiviral mediated gene correction can recover immune defects associated with XIAP deficiency, providing proof of principle for an autologous HSC gene therapy treatment approach.

## Methods

### Mice

All animal studies were approved by the Institutional Research Ethics Committee (Great Ormond Street Institute of Child Health, University College London [UCL], UK) and licensed under the Animals (Scientific Procedures) Act 1986 (Home Office, London, United Kingdom). XIAP-deficient mice (XIAP^y/−^) have been previously described [58]. Mice were housed in single ventilated cages, in pathogen-free conditions, and given ad libitum access to food and water. XIAP^y/−^ mice were bred as heterozygous, and littermates were genotyped to identify homozygous and wild-type control mice.

### Vector Constructs and Copy Number Analysis

Murine and human cell experiments were carried out by using a third-generation lentiviral vector on a pCCL backbone containing codon-optimized human XIAP cDNA driven by the spleen focus-forming virus (SFFV) promoter, internal ribosomal entry site, and eGFP or eGFP alone (SFFV-XIAP-eGFP; SFFV-eGFP). Lentiviral particles were produced, vesicular stomatitis virus glycoprotein G (VSV-G) pseudotyped and titered in HEK293T cells as previously described [59]. Lentiviral integration were quantified using a TaqMan real-time PCR assay targeting the WPRE region in the lentiviral vector and murine titin or human β-actin genomic regions in cells. A stable XIAP knockdown THP-1 (human monocytic leukemia) cell line was generated by transducing wild-type THP-1 with a pGIPZ lentiviral vector containing either a miR-30 shRNA sequence targeting XIAP (Clone 302,102), or non-silencing random control shRNA sequence (Open Biosystems). In both constructs, a co-expressed GFP cassette allowed for FACSorting transduced cells. Genomic DNA was extracted from cell pellets using a DNeasy Blood and Tissue kit (QIAGEN, West Sussex, UK) following the manufacturer’s guidelines. Average vector copy number per cell was determined by multiplex qPCR using a gBLOCK standards curve (Integrated DNA Technologies).

### Cell Cultures and Cytokine Assessments

The THP-1 (human monocytic leukemia) cell line was maintained in RPMI 1640 medium (Invitrogen, Paisley, UK) supplemented with 10% fetal bovine serum (FBS, Sigma-Aldrich, Poole, UK) and 10 μg/mL each of penicillin and streptomycin (pen/strep, Invitrogen), while HEK293T cells were maintained in 10% FBS and 1% pen/strep Dulbecco’s modified Eagle’s medium (DMEM, Invitrogen). THP-1 cells were seeded at a density of 1.0 × 10^6^ cells/well in a 12-well plate and differentiated into macrophages in the presence of 10 ng/mL phorbol 12-myristate 13-acetate (PMA) for 48 h. Cells were then washed and cultured in complete RPMI media for a further 48 h prior to NOD2/TLR/dectin-1 stimulation (See Supplemental Information). XIAP^y/−^ bone marrow-derived macrophages (BMDM) were generated as previously described [34, 60]. For BMDM stimulations, cells were harvested on day 7 and seeded at a density of 4.0 × 10^5^ cells/well in a 24-well plate. All cells were cultured at 37 °C at 5% CO2. Cytokines were measured by ELISA Ready-SET-Go kit (eBioscience, Carlsbad, CA) and Cytometric Bead Array (BD Biosciences).

### HSC Reconstitution and Curdlan Challenge

For murine reconstitution studies, lineage-negative (Lin^−ve^) cells were isolated from XIAP^y/−^ bone marrow and purified by negative selection using MACS Lineage Cell Depletion Kits (Miltenyi Biotec). Lin^−ve^ cells were transduced at a multiplicity of infection (MOI) of 75 using spinoculation (45 min at 1500* g*) and cultured in StemSpam medium enriched with 100 ng/mL mSCF, 100 ng/mL mFlt3, 25 ng/mL hTPO (all PeproTech, Rocky Hill, NJ, USA), 2% FBS and 1% pen/strep. Seventy-two hours after transduction, cells were harvested and analyzed by flow cytometry (LSRII; BD, San Jose, CA) for transduction efficiency. A colony-forming unit (CFU) assay was performed in MethoCult GF M3434 (STEMCELL Technologies, Grenoble, France). Recipient XIAP^y/−^ mice were lethally irradiated with 6 Gray (Gy) + 4 Gy in 2 consecutive days, and 5.0 × 10^5^ gene-corrected Lin^−ve^ cells/mouse was intravenously (i.v.) injected into pre-conditioned mice. Eight weeks after reconstitution, mice were challenged through intraperitoneal injections with the dectin-1 ligand curdlan as previously described [32].

### Patient Samples

Consent was obtained to use samples from 3 unrelated patient donors. All patients had confirmed mutations in XIAP (P1, age 8, exon 3 and 4 deletion; P2, age 4, c.712 C > T pArg238 stop codon; and P3, age 39, c.1396 G > T).

### Flow Cytometry, XIAP Staining and L18-MDP Assay

Single-cell suspensions were prepared from murine peripheral blood and splenic tissue. Cells were freshly stained for 30 min at room temperature with various antibodies: hamster anti-mouse CD11c APC (HL3), hamster anti-mouse CD3 APC-Cy7 (45-2C11), mouse anti-mouse NK-1.1 BV421 (PK136), rat anti-mouse CD11b PE-Cy7 or BV421 (M1/70), rat anti-mouse CD4 PerCP-Cy5.5 (RM4-5), rat anti-mouse CD45R/B220 APC (RA3-6B2), rat anti-mouse CD8 V500 (53–6.7), rat anti-mouse F4/80 PE-Cy7 (T45-2343) and, rat anti-mouse Gr-1 BV421 (RB6-8C5). The dimension reduction algorithm tSNE was applied to flow cytometry data (~20,000 cells per sample) from total splenocytes of curdlan-challenged animals using default parameters (iterations: 1000, perplexity: 30 and KNN algorithm: vantage point tree) in FlowJo™ v10.8 Software (BD Life Sciences). For human peripheral blood mononuclear cells (PBMC) isolation, red blood cell (RBC) lysis was performed with RBC lysis buffer (BioLegend, San Diego, CA, USA), and CD14^+^ monocytes were purified by positive selection using CD14 MicroBeads (Miltenyi Biotec) as per manufacturer’s guidelines. For intracellular XIAP staining, after fixation and permeabilization (IntraPrep; Beckman Coulter, Fullerton, CA), cells were incubated either with a mouse anti-human/mouse XIAP (clone 48) or the corresponding isotype control mouse IgG1, κ (both from BD Biosciences, San Jose, CA, USA). Human PBMC-derived CD14^+^ were transduced at an MOI of 20 in the presence of virion-like particles carrying Vpx (kindly provided by Giorgia Santilli). Gene-modified CD14^+^ cells were cultured for 72 h with 50 ng/mL macrophage colony-stimulating factor and analyzed by flow cytometry for transduction efficiency, and XIAP protein reconstitution. The L18-MDP assay was then carried out to determine correction of NOD2 responses in patient cells as previously described [61].

### Statistical Analysis

Statistical analysis was performed with GraphPad Prism 6.0 software (GraphPad Software, La Jolla, CA). Statistical significance for murine experiments was determine by using 2-way ANOVA. To determine statistical significance for in vitro assays, appropriate nonparametric testing was used, including Kruskal–Wallis multiple comparison tests, and Mann–Whitney 2-tailed Student *t* tests assuming non-Gaussian distribution. The significance level is shown as follows: ∗ *P* < 0.05, ∗  ∗ *P* < 0.01, ∗  ∗  ∗ *P* < 0.001, ∗  ∗  ∗  ∗ *P* < 0.0001.

### Restoration of Cytokine Defects in XIAP-Deficient Myeloid Cells

**Fig. 1:**
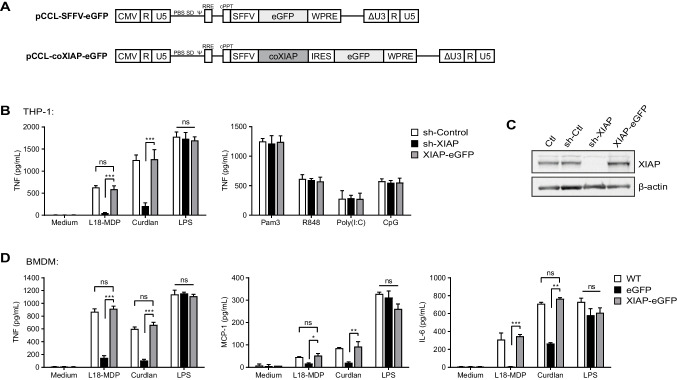
Correction of NOD2 and dectin-1 innate immune responses in myeloid cells. **A** Schematic representation of LV vectors containing human XIAP cDNA driven by the SFFV promoter and eGFP or SFFV-eGFP only. **B** PMA-differentiated THP-1 cells were treated for 2 h with NOD2/TLR/dectin-1 ligands (See Supplemental Information). ctr, control. Secreted TNF in gene-correct THP-1 macrophages. **C** A stable XIAP knockdown human monocytic cell THP-1 was established and transduced using SFFV-XIAP-eGFP or SFFV-eGFP vectors at an MOI of 20. Transduction efficiency ranged between 65 to 77% (data not shown). **D** Recovery of NOD2- and dectin-1-indiced TNF, IL-6, and MCP-1 production in gene-corrected XIAP^y/−^ BMDMs were quantified by Cytokine Bead Array. BMDMs were treated for 4 h with NOD2/TLR/dectin-1 ligands (See Supplemental Information). XIAP^y/−^ BMDMs were transduced using SFFV-XIAP-eGFP or SFFV-eGFP vectors at an MOI of 20. Transduction efficiency ranged between 50 and 84% (see Supplemental Fig. 1B), with a vector copy number of 3 to 4 copies per cell. Data are shown as means ± SEM of three independent experiments (*n* = 3 mice per group) performed in triplicates

We generated a self-inactivating lentiviral vector with a pCCL backbone that has been frequently used in gene therapy clinical trials [62, 63] containing codon-optimized human XIAP cDNA under the transcriptional control of the SFFV promoter and an eGFP reporter gene (LV-SFFV-XIAP-GFP). To investigate whether XIAP transgene expression can correct innate immune responses in myeloid cells, we transduced THP-1 XIAP knockdown cells (sh-XIAP) and XIAP^y/−^ bone marrow-derived macrophages (BMDMs) using the LV-SFFV-XIAP-GFP vector alongside a GFP control vector (LV-SFFV-GFP) (Fig. 1A). NOD2- and dectin-1-mediated inflammatory responses were successfully restored by XIAP gene transfer (Fig. 1B, C). To study cytokine responses, knockdown THP-1 cells were differentiated into macrophages in the presence of PMA and stimulated with PRR agonists. A striking reduction in TNF secretion was observed in XIAP-deficient THP-1 cells (*P* < 0.001), which was restored to control levels following transduction with the LV-SFFV-XIAP-eGFP construct (Fig. 1B; left panel). Stimulation with TLR ligands as a positive control induced TNF secretion in XIAP-deficient THP-1 cells (Fig. 1B; right panel).

Other studies have shown attenuated inflammatory responses in XIAP^y/−^ BMDMs in response to NOD2 [26, 34] and dectin-1 stimulation [32], so we next investigated whether gene-corrected BMDMs were able to efficiently regain their innate inflammatory responses in a XIAP-dependent manner. Since XIAP is important for NOD2- and dectin-1-mediated inflammatory responses, we observed reduced IL-6, MCP-1 and TNF secretion following NOD2 and dectin-1 stimulation (Fig. 1D, See Supplemental Information) but not TLR-4 stimulation in XIAP^y/−^ BMDMs as expected. Following lentiviral-mediated XIAP gene transfer, cytokine responses in gene-corrected XIAP^y/−^ BMDMs were restored to levels comparable to BMDMs from wild-type (WT) littermates. Transduction efficiency ranged between 50 and 84% (see Supplemental Fig. 1B), with a vector copy number of 3–4 copies per cell. Indeed, successful protein reconstitution in XIAP^y/−^ BMDMs using our LV-SFFV-XIAP-eGFP vector was able to restore NOD2- and dectin-1-mediated secretion of IL-6, MCP-1 and TNF compared to XIAP^y/−^ BMDMs transduced with SFFV-eGFP only (Fig. 1D, Supplemental Fig. 1A, B). These findings indicate that lentiviral gene transfer in myeloid cells can reconstitute specific innate immune responses associated with XIAP deficiency.

### XIAP Gene Transfer to HSCs Restores Innate Immune Function in an In vivo Murine Model

**Fig. 2:**
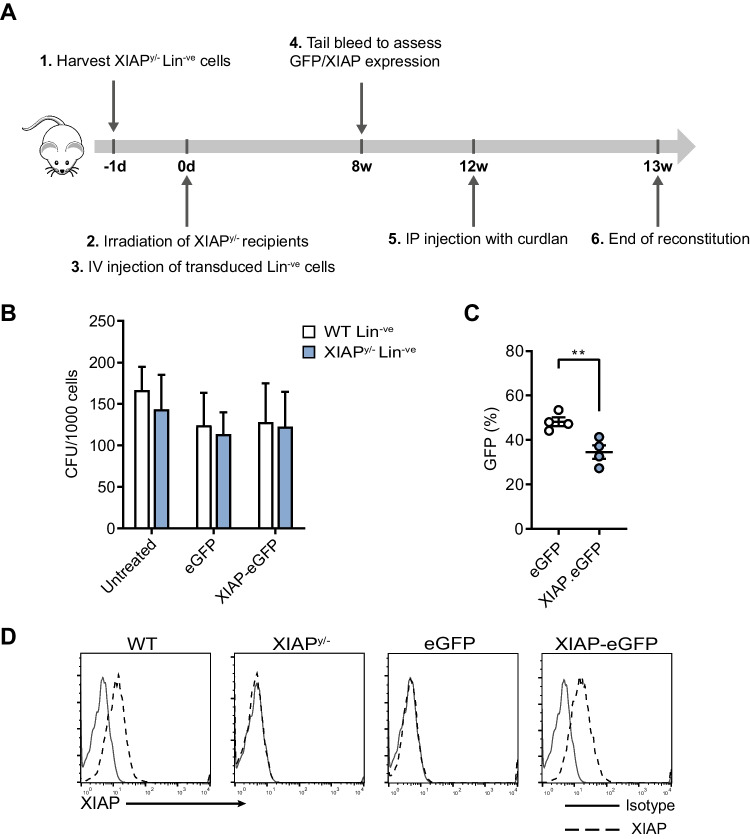
Experimental design of the gene-corrected HSC transfer and curdlan challenge model in XIAP^y/−^ mice. **A** Timeline of gene-corrected HSC transfer experiments following irradiation (6 Gy + 4 Gy in 2 consecutive days) on day 0 with intravenous infusion of murine Lin^−ve^ cells transduced with SFFV-XIAP-eGFP or SFFV-eGFP vectors at an MOI of 75. **B** Transduced Lin^−ve^ cells that were not used for transplants remained in culture for CFU assays. **C** Animals underwent tail vein bleeds at 8 weeks to assess peripheral donor cell engraftment before immunologic challenge at week 12 with the dectin-1 curdlan. Level of eGFP expression and (**D**), reconstitution of XIAP protein in PBMCs isolated from transplanted XIAP^y/−^ animals at 8 weeks, with an average vector copy number of 3 copies per cell, for both vectors (*n* = 4 mice per group). Protein expression was analyzed by using intracellular fluorescence-activate cell sorting staining. Solid line, Control IgG1κ; dotted line, anti-XIAP antibody

Priming of XIAP^y/−^ mice with the dectin-1 ligand curdlan induces features of XIAP deficiency, including compromized early innate responses to curdlan followed by elevated levels of proinflammatory cytokines which invariably result in splenomegaly and cachexia [32]. Following rescue of immune function in XIAP-deficient myeloid cells using our LV-SFFV-XIAP-eGFP vector, we next conducted in vivo studies to test the vector in an established murine model of XIAP deficiency (Fig. 2A). Bone marrow was isolated from XIAP^y/−^ donor animals and enriched for Lin^−ve^ cells, which were transduced with either the LV-SFFV-eGFP or LV-SFFV-XIAP-eGFP vector at an MOI of 75. 2 × 10^5^ gene-corrected donor XIAP^y/−^ Lin^−ve^ cells were then transferred into lethally irradiated XIAP^y/−^ recipients (transduction efficiency 51–81% for both XIAP and GFP control vectors, respectively; Supplemental Fig. 2A and B). Lentiviral-mediated gene transfer did not adversely affect colony formation (Fig. 2B).

After 8 weeks, engraftment was assessed (eGFP and XIAP expression, and vector copy number in peripheral blood) (Fig. 2C, D and Supplemental Fig. 2C) and recovery of different hematopoietic cell lineages in the periphery was similar in all irradiated animals (Supplemental Fig. 2D). The average vector copy number in PBMCs was 3 viral copies per cell, for both LV-SFFV-eGFP and LV-SFFV-XIAP-eGFP vectors. At 12 weeks after infusion of gene-corrected HSCs, XIAP^y/−^ mice were challenged with the dectin-1 ligand curdlan and analyzed at 2-h post-challenge and again 9 days later to investigate immune responses, including serum cytokines, splenomegaly and myeloid cell infiltration, as well as loss of body mass. Mice were sacrificed after 9 days and engraftment was measured by eGFP expression in blood, bone marrow, spleen, liver, and thymus (Supplemental Fig. 2E).

**Fig. 3:**
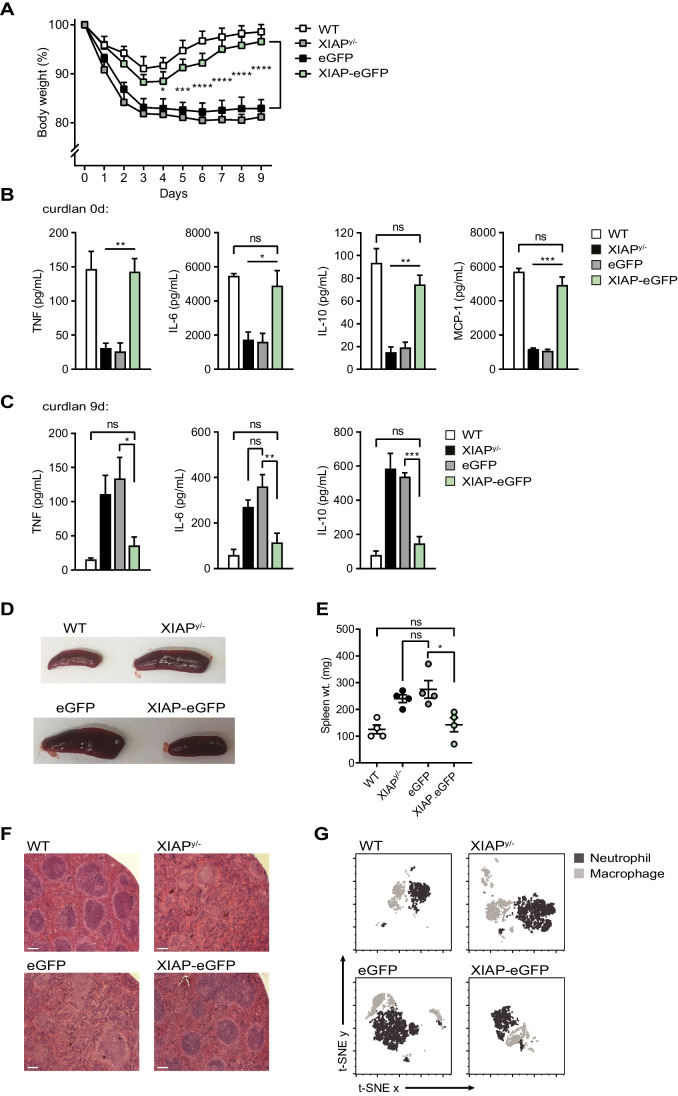
Intravenous infusion of gene-corrected HSCs from XIAP-deficient mice restores immune function in a dectin-1 ligand curdlan challenge model. **A** Recovery of body weight in XIAP^y/−^ mice transplanted with SFFV-XIAP-eGFP-transduced HSCs. Male mice (12 weeks after reconstitution) were challenged with intraperitoneal administration of curdlan (5 mg/20 g body weight), and body weight was measured daily; *n* = 4 mice per group. **B** Restored inflammatory cytokine production in gene-corrected XIAP^y/−^ animals following curdlan priming. Serum was collected via tail vein bleeding 2 h after curdlan administration, and the levels of IL-6, IL-10, MCP-1 and TNF-α was quantified and (**C**), repeated 9 days later. Increased inflammatory cytokine secretion in XIAP^y/−^ mice transplanted with SFFV-eGFP-transduced HSCs (mock vector) 9 days after curdlan challenge; *n* = 4 mice per group. **D** Gene-correction rescues curdlan-induced splenomegaly in XIAP^y/−^ mice. Spleens were harvested from all animals 9 days after intraperitoneal curdlan stimulation for size and (**E**), weight comparison. **F** Sections of spleen (4 μm) were stained with hematoxylin and eosin. One representative image from each group is shown; n = 4 mice per group. Bar indicates 50 μm. **G** Dimension reduction analysis showing macrophage and neutrophil infiltration 9 days after curdlan challenge. Representative analyses of WT, XIAP^y/−^, uncorrected XIAP^y/−^ (eGFP), and gene-corrected XIAP^y/−^ (XIAP-eGFP) animals are shown. Total splenocytes were prepared from the spleen in mice from D and stained with anti-F4/80 and anti-Gr-1. The population of macrophages (Gr-1^−/int^ F4/80^+^) and neutrophils (Gr-1^+^F4/80^−^) was determined by flow cytometry

In XIAP^y/−^ mice reconstituted with LV-SFFV-eGFP only vector, a single peritoneal administration of curdlan induced body mass loss that was not recovered by day 9 (Fig. 3A), consistent with data from Hsieh et al. [32]. WT and gene-corrected XIAP^y/−^ animals regained their body weight within a week following curdlan administration. At 2-h blood sampling, serum levels of IL-6 (*P* = 0.03), IL-10 (*P* = 0.001), MCP-1 (*P* = 0.0002), and TNF (*P* = 0.002) were reduced in XIAP-deficient mice; however, we observed significantly higher cytokines responses in WT and gene-corrected animals compared with those receiving the eGFP control vector (Fig. 3B). Splenomegaly was observed in XIAP^y/−^ mice reconstituted with the SFFV-eGFP only vector compared to WT and gene-corrected XIAP^y/−^ animals, which was associated with infiltrating neutrophils and macrophages (Fig. 3D–G). Moreover, we were able to demonstrate appropriate cessation of inflammatory cytokines in gene-corrected mice, whereas XIAP^y/−^ animals reconstituted with the SFFV-eGFP only vector developed an inflammatory cytokine profile 9 days after curdlan injection (Fig. 3C). XIAP^y/−^ mice receiving HSCs transduced with LV-SFFV-XIAP-eGFP vector exhibited weight loss, spleen size and histology and cytokine profiles comparable to WT mice. Our findings indicate that lentiviral gene therapy can recover the major defects of innate immunity in XIAP^y/−^ mice.

### XIAP Gene Transfer Restores NOD2 Innate Immune Responses in Patient Monocytes

**Fig. 4:**
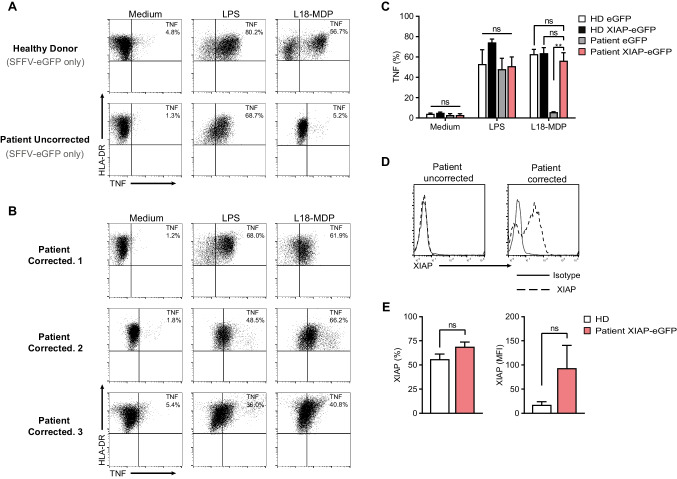
XIAP gene transfer restores NOD2 immune responses in CD14^+^ monocytes of patients with XIAP deficiency.** A** Representative flow cytometric contour plots of TNF-production in mock-transduced monocytes from healthy donors (HD) and patients (*n* = 3), and (**B**, **C**), TNF-producing monocytes from patients transduced with corrective lentiviral XIAP vector. Transduction efficiency was assessed by using flow cytometry; eGFP expression ranged from 57 to 80% (data not shown), with an average vector copy number of 1 copy per cell. Monocytes were cultured in the presence of medium only, L18-MDP (200 ng/mL) or LPS (200 ng/mL) for 2.5 h. TNF-producing monocytes were defined by forward-/side-scatter, surface expression of CD14 and human leucocyte antigen D-related (HLA-DR). The percentages indicate the fraction of TNF-positive cells of all HLA-DR^+^CD14^+^ monocytes. **D** XIAP expression in uncorrected and gene-corrected cells from patients, as analyzed by using intracellular fluorescence-activate cell sorting staining. *Solid line*, Control IgG1κ; *dotted line*, anti-XIAP antibody. **E** The Percentage (left) and mean fluorescence intensity (MFI) (right) of XIAP^+^ cells within the HLA-DR^+^CD14.^+^ monocyte population was assessed by flow cytometry in HD or patient monocytes transduced with corrective lentiviral XIAP vector (*n* = 3)

To investigate the human relevance of the data generated in the XIAP-deficient murine model, we tested whether restoration of XIAP expression in patient-derived CD14^+^ monocytes could reconstitute NOD-2-dependent innate immune function, using the functional assay previously described by Ammann et al. [61]. All patients, including two pediatric and one adult patient, were confirmed to have XIAP deficiency by Sanger sequencing and demonstrated abnormal intracellular XIAP expression. Patient CD14^+^ monocytes were isolated from PBMCs and transduced with either the LV-XIAP-SFFV-eGFP or LV-SFFV-eGFP control vector at an MOI of 20 (transduction efficiency 57–80%). Transduction with the LV-SFFV-XIAP-eGFP vector was able to restore XIAP protein expression in patient CD14^+^ monocytes, which was comparable to healthy donor monocytes as measured by intracellular staining (Fig. 4). Total XIAP-expressing monocytes in healthy donor and patient corrected cells were 56% and 69%, respectively (*P* = 0.14).

Following successful protein reconstitution, we next sought to determine if gene-corrected patient CD14^+^ monocytes regain their NOD2-dependent innate immune function in response to L18-MDP stimulations, an inflammatory response primary mediated by monocytes following NF-κB activation downstream of NOD2 [64]. TNF responses in patient-derived CD14^+^ monocytes transduced with LV-SFFV-eGFP were severely reduced in all patients (Fig. 4A), whereas gene-corrected patient monocytes transduced with LV-SFFV-XIAP-eGFP with an average vector copy number of 1 viral copy per cell demonstrated significantly higher levels of TNF^**+**^ cells in response to L18-MDP stimulation (*P* = 0.003) and are comparable to healthy controls (Fig. 4B–E). These data suggest gene-correction in CD14^+^ monocytes rescue innate inflammatory responses following NOD2 pathway stimulation. Moreover, our data supports the use of gene-correction strategies as an alternative treatment option for patient with XIAP deficiency.

## Discussion

Hematopoietic stem cell gene therapy has been used successfully to treat a number of monogenic immunological, hematological and metabolic diseases [11–16, 65–67], and offers a curative treatment option for patients lacking a suitable donor for HSCT. XIAP deficiency is an inborn error of immunity with a range of severe manifestations, and even when diagnosed early, provision of prophylactic therapies and close monitoring may not prevent fatal complications such as HLH. Outcomes following HSCT are significantly worse in patients with XIAP deficiency than those with other familial forms of HLH and even low grade GvHD is associated with disproportionate mortality [8, 9, 68]. Studies by Marsh et al. [8] and Varghese et al. [68] show high levels of toxicity following conventional myeloablative conditioning, likely due to loss of XIAP. A high incidence of GvHD, mixed donor chimerism (< 95%) and relapsed HLH post-HSCT have been observed when using RIC-HSCT [9]. As such, development of autologous gene therapy strategies can offer patients lacking suitable donors an alternative clinical option which removes any risk of alloreactivity and allows the use of reduced toxicity conditioning.

Here, we provide evidence that transfer of gene-corrected hematopoietic progenitors can correct innate immune responses associated with XIAP deficiency in a murine model, which could translate to therapeutic benefit. Further proof of concept is shown by correction of impaired immune function in CD14^+^ patient monocytes with normalization of cytokine responses following XIAP gene transfer with an average copy number of 1 viral copy per cell. This is supported by in vitro studies of XIAP-deficient THP-1 cells and BMDMs from the established XIAP^y/−^ murine model showing that gene-correction leads to rescue of both NOD2- and dectin-1-mediated cytokine responses.

Using a previously described curdlan challenge model [32], we have shown in vivo transfer of XIAP gene-corrected HSCs results in recovery of dectin-1-dependent innate immunity through specific cytokine responses, which are dysregulated in XIAP-deficient mice following curdlan administration. Early production of proinflammatory cytokines (IL-6, MCP-1 and TNF) following dectin-1 stimulation suggest the initial inability of XIAP^y/−^ mice to mount an effective innate immune response to curdlan but this was restored in gene-corrected animals. Moreover, gene-corrected animals did not demonstrate continued stimulation accompanied by accumulation of proinflammatory cytokines leading to cachexia and splenomegaly, which were markedly infiltrated by macrophages and neutrophils, unlike their non-corrected littermates. Restoration was achievable at levels of engraftment close to 40%; a clinically feasible level of correction in patients from our experience with other lentiviral gene therapy trials for inborn errors of immunity (IEI). Together, these data suggest that HSC gene therapy could be of considerable clinical benefit to XIAP-deficient patients presenting with severe inflammatory conditions such and HLH and IBD.

Our data also confirm that lentiviral-mediated XIAP gene transfer can correct innate immune defects in CD14^+^ monocytes from patients in the context of NOD2 signaling. We reliably show that gene-corrected CD14^+^ monocytes from patients can induce NOD2-mediated immune responses through ex vivo functional assays where L18-MDP-induced TNF production can be restored to healthy donor levels in patient-corrected cells (60% vs 56%, respectively) compared to only 6% response in uncorrected patient cells.

The murine model has some limitations but is still a valuable preclinical model. XIAP-deficient mice have not been shown to have a susceptibility to develop IBD or HLH like their human counterparts, but instead have compromized immunity leading to decreased survival under certain infectious conditions [2, 7]. Defects in NOD2- and dectin-1-mediated cytokine production by XIAP-deficient myeloid cells has been associated with compromized innate immunity to *L. monocytogenes* and *C. albicans*, respectively [32, 69]. Interestingly, XIAP-deficient mice challenged with MHV-68 as a surrogate for EBV infection, or *C. albicans* developed splenomegaly with increased levels of proinflammatory cytokines [32, 34]. In these models, splenomegaly occurs secondarily to the innate immune defect, which could be dependent of high levels of inflammation due to the persistence of pathogens [2].

The robust data presented here demonstrates correction of the inflammatory phenotype following gene transfer into HSCs. The cytokine profile reported in XIAP^y/−^ mice recapitulates those observed in patients [32, 34, 60], and therefore our data suggests that we could improve the pro-inflammatory status and clinical manifestations of XIAP deficiency. Although the majority of HLH in patients reported in earlier series were triggered by EBV infections [70–72], the exact mechanism for the development of HLH in XIAP deficiency is currently poorly understood but is it likely that dysregulated innate immune responses and cytokine production play a role.

For these studies, we used a lentiviral vector with the XIAP transgene under transcriptional control of the SFFV promoter, which is a viral promoter and not suitable for clinical use given the increased risk of insertional mutagenesis and clonal events with viral regulatory elements [73–76]. We investigated numerous promoter elements currently in clinical use (including MND, EFS and PGK promoters) but were unable to achieve sufficient XIAP expression levels to correct the immune defects described here. Reassuringly, we did not observe any detrimental effects of XIAP ‘overexpression’ in murine HSC function and hematopoietic development, but experiments were not specifically designed to assess tumorgenicity. While this data serves as proof of concept that corrective gene transfer ameliorates the immune abnormalities seen in this condition, with the highly regulated expression profile of XIAP in immune lineages, achieving as close to a physiological expression profile will be crucial. Thus, we are now focusing our efforts on targeted gene editing platforms to precisely correct the XIAP gene in situ, allowing expression from endogenous regulatory elements which should improve the safety and efficacy of gene therapy for this disease. The level of correction required to ameliorate disease phenotype will only truly be understood through clinical studies. There are reports describing female carriers with random X inactivation experiencing mild inflammatory symptoms [3, 77]; however, we are encouraged that the high levels of correction (> 50%) resulted in restoration of NOD2 signaling, suggesting that this level is sufficient to provide clinically relevant benefit.

In conclusion, the data shown here strongly supports the development of gene-correction approaches to treat patients with XIAP deficiency and overcome complications associated with HSCT.

## Supplementary Information

Below is the link to the electronic supplementary material.

Supplementary file 1 (DOCX 18 KB)

Supplementary file 2 (PDF 157 KB)

## Data Availability

Not applicable.
