# Supplementary material for: Lentiviral Gene Transfer Corrects Immune Abnormalities in XIAP Deficiency
Source: J Clin Immunol. 2022 Nov 3;43(2):440–51. doi: 10.1007/s10875-022-01389-0 (PMC9892131; doi:10.1007/s10875-022-01389-0)
Supplement: Supplementary file 1 — (DOCX 18 KB) [file 10875_2022_1389_MOESM1_ESM.docx]

**Supplemental information**

**Reagents:** CpG (Invivogen), curdlan (Peprotech), human/murine M-CSF (Peprotech), L18-MDP (Invivogen), Pam_3_CSK_4_ (Invivogen), poly (I:C; Invivogen), GolgiPlug^TM^ (BD Biosciences), R848 (Invivogen), red cell lysis buffer (eBioscience), ultrapure LPS (Invivogen). All antibodies for surface marker staining experiments were obtained from BD Biosciences.

**NOD2/TLR/dectin-1 stimulations:** THP-1 cells were differentiated in the presence of PMA and treated for 2 hours with L18-MDP (200 ng/mL), curdlan (10 μg/mL), LPS (100 ng/mL), Pam_3_CSK_4_ (400 ng/mL), R848 (1 μg/mL), poly (I:C; 25 μg/mL), and CpG (2 μg/mL). BMDMs were treated for 4 hours with LPS (200 ng/mL), L18-MDP (1 μg/mL) or curdlan (100 μg/mL).

**Supplemental Figure Legends**

**Fig. S1** Reconstituted XIAP in BMDMs transduced *ex vivo* with lentiviral vectors. (**A**) XIAP^y/-^ BMDMs were transduced with SFFV-XIAP-eGFP or SFFV-eGFP vectors at an MOI of 20 and protein expression was confirmed, as analyzed by using intracellular fluorescence-activate cell sorting staining. *Solid line*, Control IgG1κ; *dotted line*, anti-XIAP antibody. (**B**) Transduction efficiency was assessed by using flow cytometry; eGFP expression ranged from 50% to 84%. UT, untransduced.

**Fig. S2** Reconstituted XIAP in donor Lin^-ve^ cells transduced *ex vivo* with lentiviral vectors (**A**) XIAP^y/-^ donor Lin^-ve^ cells were transduced with SFFV-XIAP-eGFP or SFFV-eGFP vectors at an MOI of 75 and protein expression was confirmed, as analyzed by using intracellular fluorescence-activate cell sorting staining. *Solid line*, Control IgG1κ; *dotted line*, anti-XIAP antibody. (**B**) Transduction efficiency was assessed by using flow cytometry; eGFP expression ranged from 51% to 81%. UT, untransduced. (**C**) The Percentage (left) and mean fluorescence intensity (MFI) (right) of XIAP protein in PBMCs isolated from WT and transplanted XIAP^y/-^ animals at 8 weeks (n = 4). (**D**) Flow cytometric analysis of haematopoietic lineages in control and experimental mice 8 weeks after reconstitution with gene-modified Lin^-ve^ cells. (**E**) Level of eGFP expression in the blood, bone marrow, spleen, liver, and thymus of all animals at the time of sacrifice.
