# Supplementary figures and images for: Lentiviral Gene Transfer Corrects Immune Abnormalities in XIAP Deficiency
Source: J Clin Immunol. 2022 Nov 3;43(2):440–51. doi: 10.1007/s10875-022-01389-0 (PMC9892131; doi:10.1007/s10875-022-01389-0)

Figure S1

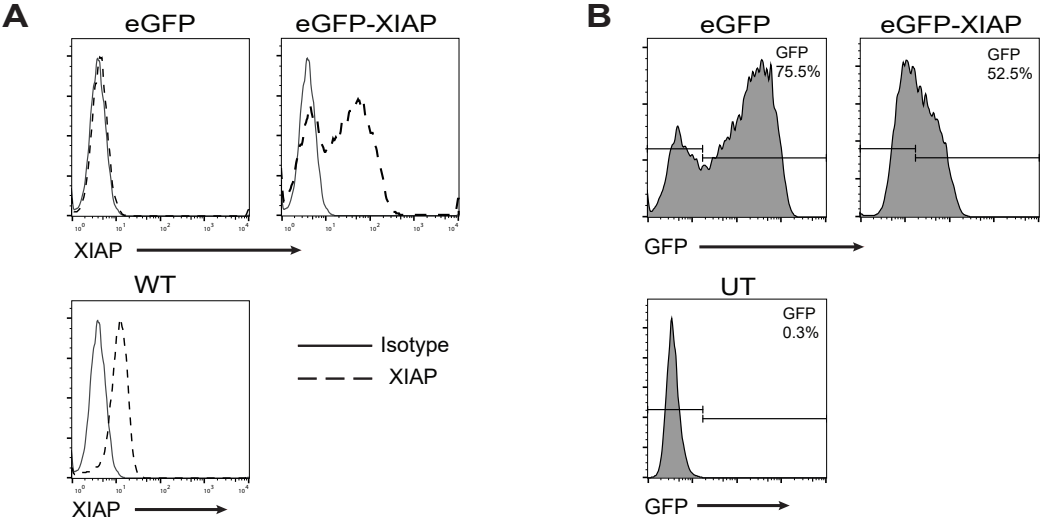

**Figure S2**

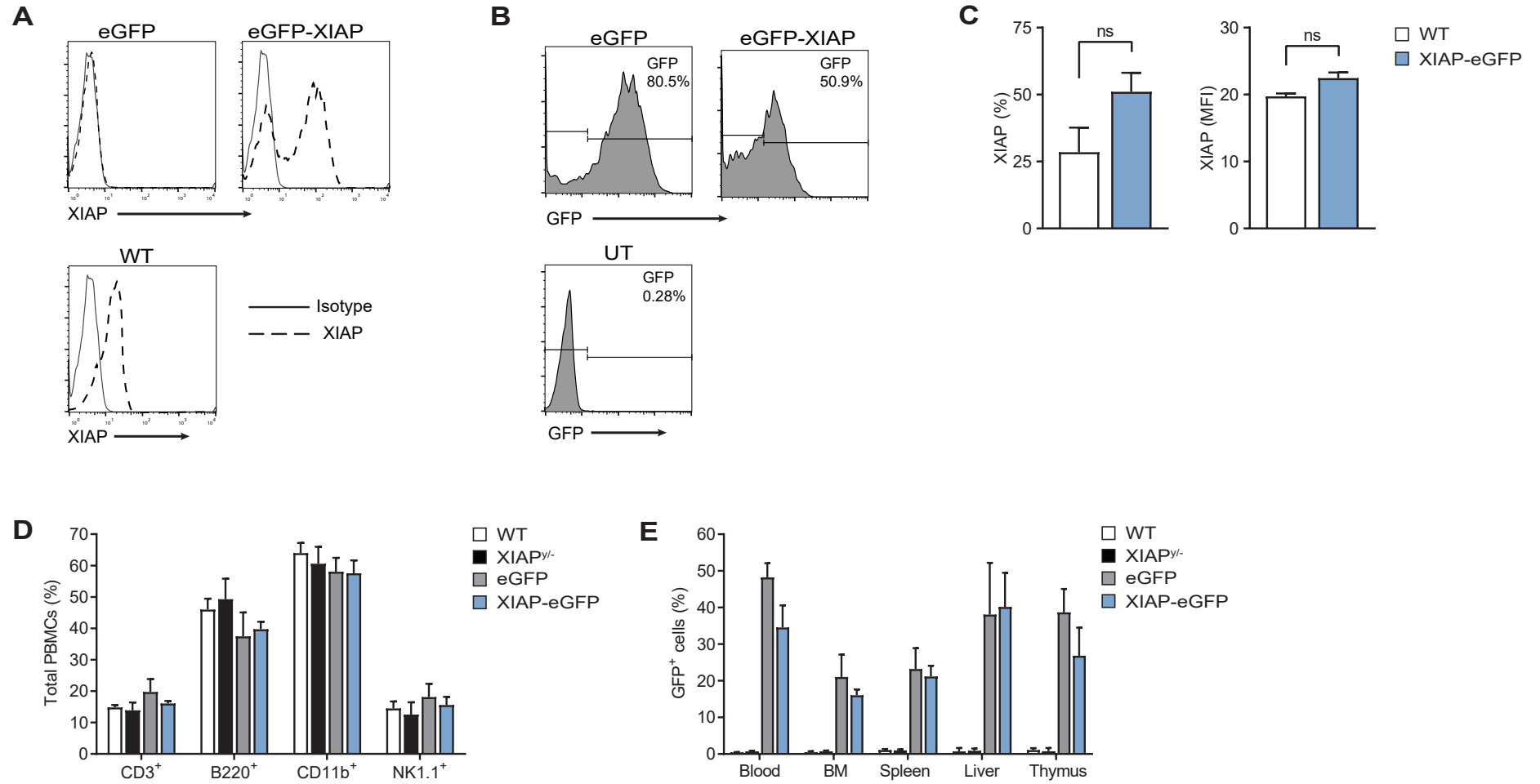

Supplement: Supplementary file 2 — (PDF 157 KB) [file 10875_2022_1389_MOESM2_ESM.pdf]
